# Latent Dirichlet Allocation in predicting clinical trial terminations

**DOI:** 10.1186/s12911-019-0973-y

**Published:** 2019-11-27

**Authors:** Simon Geletta, Lendie Follett, Marcia Laugerman

**Affiliations:** 1 Department of Public Health, Des Moines University, 169 Ryan Hall, 3200 Grand Ave, Des Moines, IA USA; 2 Department of Data Analytics, College of Business and Public Administration, Drake University, Des Moines, IA USA

**Keywords:** Clinical trials, Structured data, Unstructured data, Latent Dirichlet allocation, Prediction

## Abstract

**Background:**

This study used natural language processing (NLP) and machine learning (ML) techniques to identify reliable patterns from within research narrative documents to distinguish studies that complete successfully, from the ones that terminate. Recent research findings have reported that at least 10 % of all studies that are funded by major research funding agencies terminate without yielding useful results. Since it is well-known that scientific studies that receive funding from major funding agencies are carefully planned, and rigorously vetted through the peer-review process, it was somewhat daunting to us that study-terminations are this prevalent. Moreover, our review of the literature about study terminations suggested that the reasons for study terminations are not well understood. We therefore aimed to address that knowledge gap, by seeking to identify the factors that contribute to study failures.

**Method:**

We used data from the clinicialTrials.gov repository, from which we extracted both structured data (study characteristics), and unstructured data (the narrative description of the studies). We applied natural language processing techniques to the unstructured data to quantify the risk of termination by identifying distinctive topics that are more frequently associated with trials that are terminated and trials that are completed. We used the Latent Dirichlet Allocation (LDA) technique to derive 25 “topics” with corresponding sets of probabilities, which we then used to predict study-termination by utilizing random forest modeling. We fit two distinct models – one using only structured data as predictors and another model with both structured data and the 25 text topics derived from the unstructured data.

**Results:**

In this paper, we demonstrate the interpretive and predictive value of LDA as it relates to predicting clinical trial failure. The results also demonstrate that the combined modeling approach yields robust predictive probabilities in terms of both sensitivity and specificity, relative to a model that utilizes the structured data alone.

**Conclusions:**

Our study demonstrated that the use of topic modeling using LDA significantly raises the utility of unstructured data in better predicating the completion vs. termination of studies. This study sets the direction for future research to evaluate the viability of the designs of health studies.

## Background

Several recent studies have reported a conspicuous prevalence of termination of studies that are conducted with full financial and institutional support from national agencies such as the National Science Foundation (NSF), National Institute of Health (NIH), etc. One such study reported that about 19% of all studies registered on the clinicaltrials.gov repository were terminated before yielding results.

Prior to the publication of this report, Kasenda et. al., [1] followed a cohort of randomized controlled trials that were conducted in Switzerland, Germany, and Canada over a three-year period (from 2000 to 2003) and reported that about 25% of the studies that they observed were discontinued. The risk of study failure is known to vary by the study’s focus area. For example, it is reported that 19% of studies conducted between 2008 and 2010, that focused on pediatric medicine topics did not yield results. Jamjoom et al. [2] reviewed neurosurgery trials data from the ClinicalTrials.org repository and reported that about 26.6% of such trials were discontinued early. If we judge study success by publication and not termination, it is intuitively clear that the proportion of study failures is even higher than what is reported in the above literature.

In most cases of study terminations, the reasons for the terminations were not readily given. Among the known reasons for termination, inadequate subject enrollment appears to be the most common. Other factors such as unanticipated adverse drug events such as toxicity among drug trials, and early termination due to higher than expected treatment efficacy are also cited, but to a much lesser degree.

We know that scientific studies in all disciplines are initiated with extensive planning and deliberation, often by a highly-trained team of scientists. Further, to assure that the quality, integrity and feasibility of funded research projects meet certain standards, research funding agencies such as the National Institute of Health, the National Science Foundation etc., approve proposed research plans and/or proposals through a rigorous peer review process to make decisions about whether or not the projects should proceed. The proposal review process has been described as a time consuming and costly enterprise. Yet, some studies pass through all the rigorous scrutiny of the peer review process and end up being terminated before yielding results. Our assessment of this circumstance convinces us of the need to explore an approach that could be used to ameliorate the screening process so as to minimize trial terminations.

The existence of the clinicaltrials.gov repository presents a unique opportunity to study a number of issues regarding the lifecycle of scientific studies. The origin of this repository is linked back to the Food and Drug Administration Modernization Act (FDAMA) of 1997, which included the requirement to register all trials testing the effectiveness of investigational drugs for serious or life-threatening conditions. In 2000, Congress authorized the creation of the ClincialTrials.gov (CT.gov) registry to provide information and access to clinical trials for persons with serious medical conditions.

The Food and Drug Administration Amendments Act 2007 (FDAAA), established mandates requiring sponsors of applicable interventional human research studies to register and report basic summary results on CT.gov – widening the inclusiveness of studies that must be registered. In general, this included all non-phase 1 interventional trials of drugs, medical devices, or biologics initiated after September 27, 2007. The FDAAA also required that all such trials report the results within 1 year after the primary completion date or within 1 year after the date of early termination. About the same time the Health and Human Services (HHS) established a new regulation known as “the final rule” which clarified the requirements for reporting of summary results in ClinicalTrials.gov repository [3]. Currently, government funded studies that are conducted within the United States must be registered by law and as a prerequisite for publication, making CT.gov useful for cross disciplinary analysis of trends in clinical trial protocol and conduct. Although FDAAA and the HHS policies that are outlined above have improved the completeness of the clinicalTrials.gov repository data, as Cahan and Anand [4] observe, major inconsistencies in the data, resulting from the manner in which the data are reported cause significant problems for researchers who wish to use the data for analysis. Such inconsistencies create obstacles to using the “structured data” in the repository for statistical modeling and analysis.

At the time of writing this manuscript, there were 281,648 research studies registered in the clinicaltrials.gov registry, with slightly varying details about the studies. Researchers can provide information about studies in a total of 356 attribute fields, most of which are stored in the form of structured attribute data (string, numeric and date types). There are 36 fields that represent free-text fields in which lengthier descriptions of study characteristics are saved.

Recently, researchers have highlighted the ubiquity of unstructured data generated through health care practice transactions. Such observations have spurred increased interest in the application of text mining approaches in the field of health care and medical research. Examples of studies that used text mining approaches include works by Lazard, and Glowacki and collaborators [5]. Both of these researchers used a text mining approach to extract distinct topics that are present in tweets concerning health issues. Lazard et al. highlighted the use of e-cigarettes while [6, 7] focused on the public interest and concern regarding Ebola and Zika, respectively. Topic generation in text mining uses one of two approaches. The first one called “Latent Sematic Indexing” (LSI) uses the method of linear algebra (singular value decomposition) to identify topics.

The second approach, called latent Dirichlet allocation (LDA), uses a Bayesian approach to modeling documents and their corresponding topics and terms. The goal of both techniques is to extract semantic components out of the lexical structure of a document or a corpus. LDA is a more recent (and more popular) of the two approaches. It is introduced by [8] in a work that they published in 2003.

LDA uses Bayesian methods in order to model each document as a mixture of topics and each topic as a mixture of words. The word ‘mixture’ here entails a set of elements (topics or words) with corresponding probabilities. It promotes the idea that, realistically, a body of text (a document or corpus) will incorporate multiple themes and that the topics will be fluid in nature. Thus, each document can be represented by a vector of topic probabilities while each topic can be represented by a vector of word probabilities.

The number of topics used in LDA is a user supplied parameter and there currently is not a formal way of determining how many topics should be extracted using the LDA approach. Hence, researchers are generally at liberty in selecting n topics out of however many mixtures of corpora and terms they work with. Most current literature suggests that researchers in diverse applied and scientific fields are in pursuit of a suitable approach for determining the number of topics that are robust for characterizing corpora. Amado et al., in their comprehensive study of current trends on big data in marketing literature, use a simple approach suggested by [10]. However, others take a more exploratory approach and try multiple numbers of topics. Cai et al. presented an alternative way to represent documents as vectors calculated using the word-topic probabilities in conjunction with word-document counts. Cai et al. demonstrated this method using 4, 8, 12, 16, and 20 as the number of topics. They showed in an empirical study that this “probability sum” representation results in more efficient document classification.

While LDA is useful in the context of description alone, it can also be used in conjunction with supervised machine learning techniques and statistical algorithms in order to make predictions. The topic-document probabilities (or, as would be suggested by [11], probability sums) can be used as supplemental structured data as an input to prediction algorithms. For example, [12] use LDA along with AdaBoost in order to predict whether or not a body of text was derived from a phishing attempt. They demonstrated a high level of accuracy in classifying a document as a phishing attempt when it truly was a phishing attempt. Xiao et. al., [13] recognized the advantage to the interpretability of LDA results as well as its ability to increase the prediction performance of standard methods. In their work, they predict adverse drug reactions using output from LDA by using the “drug document” as the textual input. Here, topics had the useful interpretation of biochemical mechanisms that link the structure of the drug to adverse drug reactions.

Unstructured text is an integral part of the funding and acceptance of clinical trials. When a study is initially proposed, the researchers must specify expected/planned features such as enrollment numbers, enrollment requirements, assignment of treatments, timeline, and so on. Further, the researchers submit a description of the study and its ultimate research objectives. This description can contain a wealth of useful information that are used not only for funding decisions but also to investigate the studies’ life cycle. In particular, this description may very well hold the key to the intricate underlying causes of study failure or success. We propose using LDA to extract topics from the descriptions of the research studies registered in the clinicaltrials.gov. We then propose to use these topics to train a random forest to predict whether or not a trial will ultimately terminate.

Our specific goal in this study is to continue to investigate the question of to what extent study terminations can be predicted from the characteristics assigned to them prior to their funding or approval. This builds upon the work of [14]. By way of achieving this goal, we opted to fulfill the following specific objectives. First, we aim to explore the use of LDA to extract topics from the descriptions of trials prior to their funding. Second, we use the LDA-derived topic probabilities assigned to each clinical trial in order to improve the detection of trial termination over the use of standard structured data alone.

## Data

We obtained data on 252,847 studies that contained non-missing data for the “brief summaries” text field from the Clinical Trials Transformation Initiative (CTTI) through September 29, 2017. CTTI achieves data from ClinicalTrials.gov, by restructuring it in such a manner that made it suitable for statistical analyses and made it available to the public. Table 1 below provides summary information regarding the available structured variables. The variables include primary purpose of the study – a nominal scale variable that characterizes the studies by the purpose for which they are being carried out - intervention type, study phase, intervention model, allocation and enrollment (number of participants). As could be seen from this univariate summary, most the studies are completed. Radiation studies have the highest proportion of terminating (22%). Biological and behavioral studies are least likely to terminate (although there are only eight studies representing those that are labeled “biological” in terms of type.

**Table 1:** Summary of clinical trial termination rates within levels of each structured variable

Characteristic	Level	total_n	Completed	Terminated
Primary Purpose	Missing	29484	0.94	0.06
Primary Purpose	Basic Science	4915	0.94	0.06
Primary Purpose	Device Feasibility	52	0.83	0.17
Primary Purpose	Diagnostic	3858	0.87	0.13
Primary Purpose	Educational/Counseling/Training	118	0.89	0.11
Primary Purpose	Health Services Research	1971	0.96	0.04
Primary Purpose	Other	795	0.91	0.09
Primary Purpose	Prevention	11939	0.93	0.07
Primary Purpose	Screening	723	0.93	0.07
Primary Purpose	Supportive Care	3491	0.91	0.09
Primary Purpose	Treatment	76625	0.88	0.12
Intervention Type	Behavioral	10147	0.97	0.03
Intervention Type	Biological	7770	0.90	0.10
Intervention Type	Combination Product	8	1.00	0.00
Intervention Type	Device	10957	0.88	0.12
Intervention Type	Diagnostic Test	73	0.96	0.04
Intervention Type	Dietary Supplement	3811	0.95	0.05
Intervention Type	Drug	65364	0.88	0.12
Intervention Type	Genetic	500	0.90	0.10
Intervention Type	Other1	11302	0.93	0.07
Intervention Type	Procedure	8908	0.89	0.11
Intervention Type	Radiation	751	0.78	0.22
Study Phase	Early Phase 1	915	0.88	0.12
Study Phase	Missing1	54082	0.93	0.07
Study Phase	Phase 1	17396	0.91	0.09
Study Phase	Phase 1/Phase 2	4432	0.84	0.16
Study Phase	Phase 2	22544	0.85	0.15
Study Phase	Phase 2/Phase 3	2423	0.87	0.13
Study Phase	Phase 3	17992	0.89	0.11
Study Phase	Phase 4	14187	0.90	0.10
Intervention Model	Missing2	27542	0.93	0.07
Intervention Model	Crossover Assignment	11597	0.95	0.05
Intervention Model	Factorial Assignment	1836	0.94	0.06
Intervention Model	Parallel Assignment	61036	0.90	0.10
Intervention Model	Sequential Assignment	49	0.90	0.10
Intervention Model	Single Group Assignment	31911	0.86	0.14
Allocation	Missing3	45626	0.90	0.10
Allocation	Non-Randomized	14325	0.88	0.12
Allocation	Random Sample	40	0.93	0.07
Allocation	Randomized	73980	0.91	0.09
Enrollment Group	*>* 1000	7151	0.96	0.04
Enrollment Group	0-100	80755	0.87	0.13
Enrollment Group	101-1000	42683	0.94	0.06
Enrollment Group	Missing4	3382	0.95	0.05

The trial description field is a free-text field that briefly describes the study. This field varies in length; the shortest description consists of a single word while the longest description is 822 words long. The median length of a description is approximately 54 words long. An example of one observation from the description field is as follows.

*“To determine whether radial keratotomy is effective in reducing myopia. To detect complications of the surgery. To discover patient characteristics and surgical factors affecting the results. To determine the long-term safety and efficacy of the procedure.”*

In the following section, we detail how we take free text like the above and use LDA in order to convert into numeric fields that help us understand a trial better and, ultimately, predict whether or not it terminates.

## Methods

We took a subset of the data described above to only include studies which started prior to May 1, 2015, in order to give the trials time to terminate or complete successfully. Because the criterion variable for this study is whether or not a study was completed successfully or whether it was terminated, we also subset the data to only include studies which have either been completed or terminated. The data preparation process involved joining data that are stored in separate tables in the CTTI repository using the unique clinical trial identifier. After joining and sub-setting, the data contained the the structured variables (see Table 1) and unstructured text descriptions of a total of 119,591 studies.

**Fig. 1:**
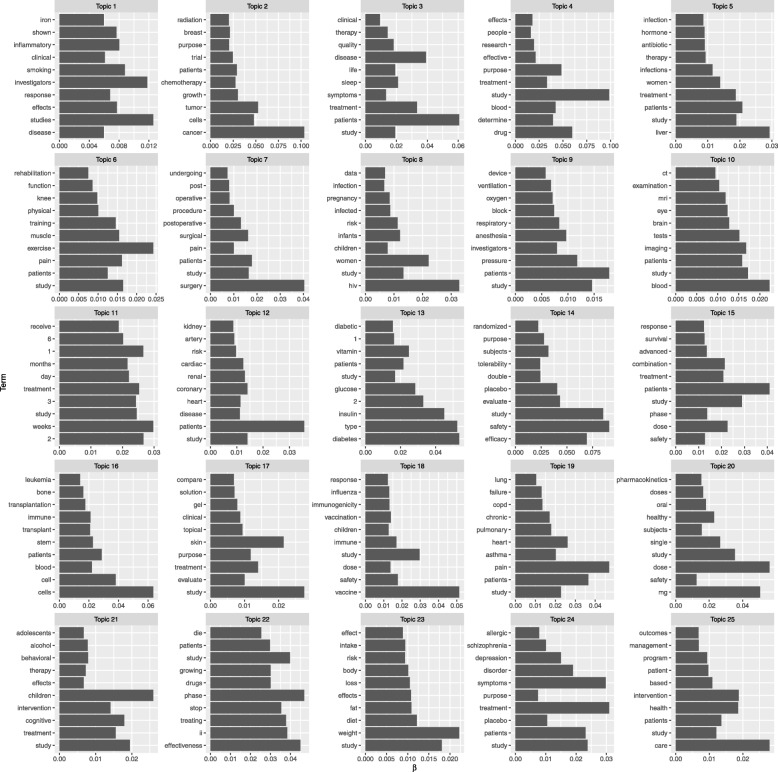
Flowchart description - this is an updated one that reflects the LDA analysis and 3 competing models

In preparing the data described above for analysis, we followed a standard workflow of text analysis. This is detailed in Fig. 1. The first step was to tokenize the free text field, transforming the data so that there is one line per word (“token”) per clinical trial description. Then, standard English “stop words” were used to eliminate tokens that do not represent meaningful aspects of language parts (e.g., “is”, “a”, “the” etc.). We use a stop word dictionary consisting of 1149 unique words derived from three lexicons which are deemed undesirable for meaningful analysis. More details on this process are given in [14].

After the text descriptions are tokenized and scrubbed of stop words, the appearance of each remaining word is counted for each trial and the result is stored in a data table from which the document-term matrix is created. The LDA is applied to the document-term matrix to create topic-word probabilities (known as the *β* matrix) and document-topic probabilities (known as the *γ* matrix) that are used in this project. We experimented with different numbers of topic probabilities before we settled on 25 topics. The decision to stop at 25 topic probabilities was partially arbitrary but was also motivated by (1) the computational cost of adding topic probabilities beyond 25 and (2) the perceived lack of definition of topics when the number exceeded 25.After extracting the 25 document-topic probabilities we then employed them as predictors in a random forest with the goal of predicting clinical trial failures. In addition to the 25 document-topic probabilities, we use the six structured variables outlined in Table 1. We use mean decrease in accuracy (see [15] for details on mean decrease in accuracy in random forest models) to assess importance of the LDA topic probabilities and structured variables in terms of predicting trial termination. Thus, our assessment will involve the fitting of two random forests: (1) where the only predictor variables are the six structured variables and (2) where we use both the structured variables and the 25 document-topic variables. This framework will allow us to assess the marginal contribution of the topic probabilities in explaining trial termination, relative to a model using only structured data. We followed the standard practice of randomly partitioning the data into 70% training and 30% model testing before the random forest predictive models were built.

Finally, the random forest is used to inform a parsimonious logistic regression model that lends estimates of directional effects of the topics in terms of how they effect the probability of termination. This logistic regression model is fit with glm using standard maximum likelihood estimation.

## Results

**Fig. 2:**
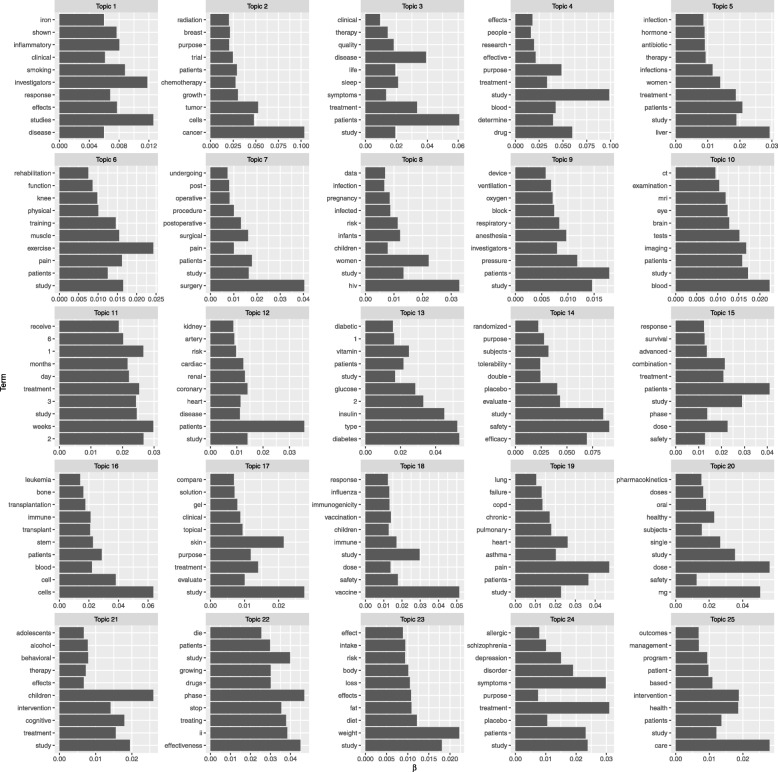
The 25 topics with the top 10 term-topic probabilities

We begin with a discussion of the topics extracted by LDA. Interpretability is a valuable quality of LDA as it applies to prediction problems because one can associate a topic of discussion with the risk of the outcome. One can use the *β* matrix to first gain an understanding of what kind of topics appear in the corpora by looking at the words which most strongly represent the topic. Figure 2 presents the 25 topics that are retained through the application of LDA. For each topic, we present the top 10 words in terms of the term-topic probabilities (*β*). Note that the probability axis varies by topic. The topics can be viewed as underlying constructs measured by the combination of terms that form the topics through probabilistic logic. For example, if we look at topic 7, it contains terms such as “surgery”, (noun), “surgical” (adjective), “postoperative” (adjective), “pain” (verb) - all of which point to the underlying construct or concept of studies that are focused on surgical procedures. Similarly, topic 17 combines verbs such as “compare” and “evaluate” with an adjective like “topical” and nouns such as “solution”, “gel”, “skin” and “treatment” suggesting the underlying construct of studies in dermatology. Such patterns can clearly be identified by inspecting each of the LDA topics. We have assigned labels to each of the 25 topics that will be used henceforth to aid in interpretability of results. These labels can be seen in Table 2.

**Table 2:** Topics, terms and possible construct descriptors

Topic	Partial words (Ordered by probability)	Construct	Rank in prediction
Topic 1	Studies, investigators, inflammatory, smoking, effects	Inflammation	7*th*
Topic 2	Cancer, tumor, cells, growth	Cancer	
Topic 3	Patients, disease, treatment, sleep, quality, therapy	Disorder	
Topic 4	Study, drug, purpose, blood, determine,	Drug	
Topic 5	Liver, patients, study, treatment	Liver	
Topic 6	Exercise, study, muscle, training	Exercise	
Topic 7	Surgery, patients, study, surgical, postoperative	Surgery	1*st*
Topic 8	HIV, women, study, infants, risk	HIV/Pregnancy	3*rd*
Topic 9	Patients, study, pressure, anesthesia, respiratory	Respiratory	9*th*
Topic 10	Blood, study, imaging, tests	Blood/Brain	5*th*
Topic 11	Weeks, months, time intervals	Duration	
Topic 12	Patients, coronary, renal, cardiac, heart, disease	Coronary	4*th*
Topic 13	Diabetes, type 1 and 2, insulin	Diabetes	10*th*
Topic 14	Safety, study, efficacy, evaluate, placebo	Safety/Efficacy	
Topic 15	Patients, study, dose, combination, treatment	Drug Dose/Combination	
Topic 16	Cell, stem cells, transplant, immune	Stem Cell	8*th*
Topic 17	Study, skin, treatment, purpose, topical	Dermatological	2*nd*
Topic 18	Vaccine, study, immune, safety, dose	Vaccine	
Topic 19	Pain, heart, pulmonary, chronic, pulmonary	Pain/Pulmonary	
Topic 20	Milligram, dose, study, single, healthy	Dose	
Topic 21	Children, cognitive, treatment, intervention	Children/Cognitive	
Topic 22	Phase, effectiveness, treating, stop	Study characteristics	
Topic 23	Weight, study, diet, fat, effects	Weight control	
Topic 24	Symptoms, treatments, disorder, depression	Mental Health	
Topic 25	Care, intervention, health, patients, management	Public Health	

**Fig. 3:**
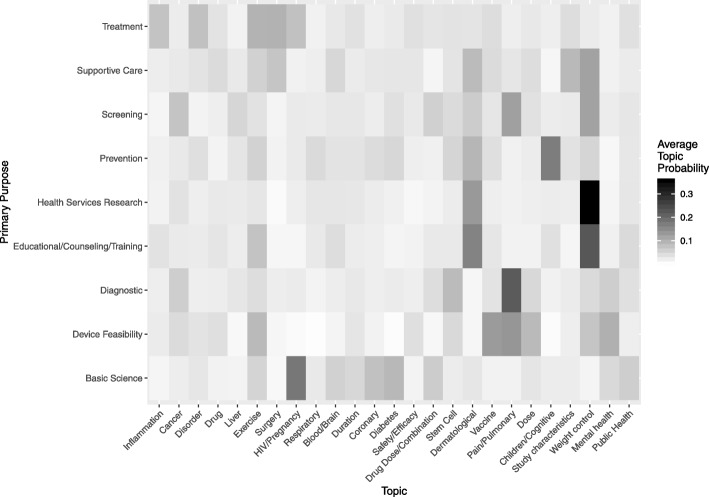
Average topic probabilities for each primary purpose category

The topic probabilities assigned to each trial provide information that supplements what is provided by the original structured variables. The LDA framework models the clinical trial description as a continuous mixture of multiple topics. Contrast this with a categorical variable such as “primary purpose” which will plant a clinical trial firmly in a single category such as “Basic Science” or “Screening”. Figure 3 illustrates this difference. In this figure, colour represents the average topic probability for trials that are assigned to each of the primary purpose categories. For example, trials that are considered “Basic Science” have a relatively high probability for topic 8 which seems to concern women and HIV as well as a high probability for topic 13, which concerns diabetes. Thus, the LDA provides additional context *within* levels of the structured variables. For this reason, LDA topic probabilities enhance predictions as well as understanding of what drives clinical trial termination.

**Fig. 4:**
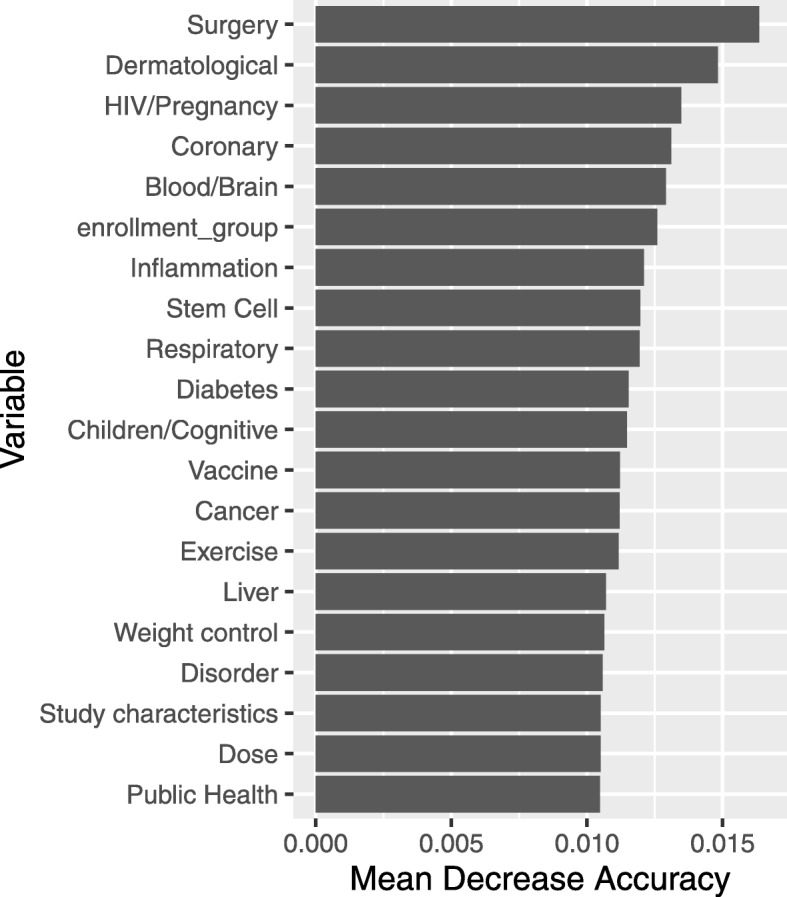
Variable importance in terms of mean decrease in accuracy for the random forest fit using all 25 LDA-generated topics as well as the standard structured variables

One way to extract meaning from a random forest model is to examine variable importance measures. Figure 4 shows the top ten variables in terms of the mean decrease in accuracy they contributed to the random forest model. This is a measure of how much the prediction accuracy of the individual trees within the forest suffers, on average, when a permuted version of the variable is used for out of bag prediction. Topic 7, which we identified as being focused on surgical procedures, appears to be the most useful in terms of increasing prediction accuracy. Topic 7 is closely followed by topic 17 the dermatology topic, the heart condition topic, and a topic concerning pregnant women and HIV. Enrollment size (an ordinal scale variable representing the number of subjects recruited by a study) ranked 5*th* in terms of importance. We note that this is the only structured variable that appears in this list of variables contributing to increased prediction accuracy.

Overall, the top four predictors in terms of mean decrease accuracy are the topic probabilities that are derived using LDA. We note that when using binary term indicators alone, as in [14], those indicators do not excel in terms of variable importance. Labeling these constructs is an important but unfortunately subjective process. Under ideal circumstances, labeling the constructed should use a “consensus” (qualitative research) approach to examine the meaning the terms. For this research, we provide a list of the topics with corresponding labels and their ranks in the random forest predictive model that was used to predict study completion or termination. Table 2 contains that information.

**Fig. 5:**
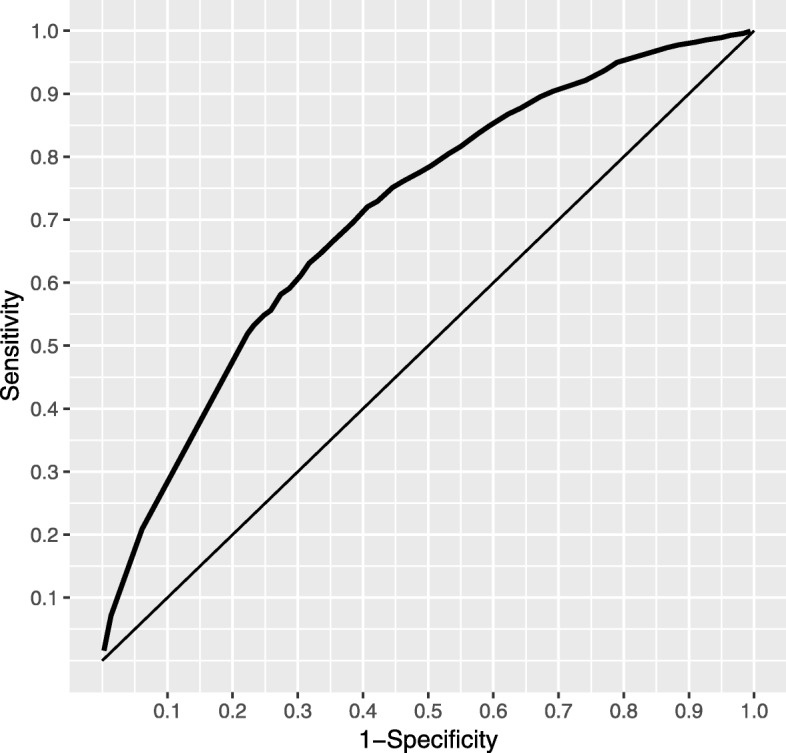
ROC curve for random forest that includes both structured variables and LDA topic probabilities

We test this trained random forest on the remaining 30% of the original data. As trial termination is a relatively rare event, we focus on an ROC curve which presents the results in terms of sensitivity and specificity; this is shown in Fig. 5. The straight line represents the ROC curve for a model that predicts at random. One can chose a threshold that results in a reasonable sensitivity and specificity, depending on the practical losses incurred by the two types of prediction error. From this ROC curve, we can see that we are able set the threshold as to obtain a sensitivity of 0.6 while still maintaining a fairly low 1-specificity of 0.3. If we raise the 1-specificity to 0.5 the sensitivity of the test will actually climb to around 0.8.

To compare the performance of a *structured variables only* model, we also run a random forest model with only the structured variables as predictors. Using only these variables, we are unable to obtain a sensitivity greater than 0.05 for any reasonable threshold and, thus, do not display a corresponding ROC curve. It appears that structured data alone is simply not granular enough to grasp the rare event of a terminated trial.

The random forest model is beneficial because it gives us a way to rank clinical trials in terms of their termination risk. In addition, we are able to assess what topics contribute most to the prediction accuracy. What we are thus far missing, however, is directional effects attributed to the most important variables; that is, whether they are associated with a higher or lower probability of termination. Using the importance information generated by the random forest, we can build a parsimonious logistic regression model in order to gain that insight. That is, if we allow

$$ {y}_i=\left\{\begin{array}{c}1\  if\ clinical\ trial\ i\  terminated\\ {}0\  if\ clinical\ trial\ i\  completed\end{array}\right. $$

we can specify a proposed model as

$$ {y}_i\sim Bernoulli\left({\pi}_i\right) $$

$$ \log \left(\frac{\pi_i}{1-{\pi}_i}\right)={\eta}_i $$

where *π*_*i*_ represents the probability of the *i*^*th*^ trial being terminated and *η*_*i*_ is the linear predictor containing the most important variables. We estimated this model using the R function glm (available in base R) using topics 7 (Surgery), 17 (Dermatological), 12 (Coronary), and 8 (HIV/Pregnancy), along with a categorical enrollment indicator. Table 3 displays the odds ratios corresponding to each variable as well as corresponding 95% profile likelihood confidence intervals. We find that higher probabilities of topic 7 (Surgery) are associated with higher termination probabilities. In particular, an increase in a topic 7 probability of 0.10 increases the odds of termination by a factor of 1*.*90^0*.*10^ = 1*.*066 (by almost 7%). On the other hand, higher probabilities of topics 17 (Dermatological), 12 (Coronary), and 8 (HIV/Pregnancy) are associated with lower termination probabilities. As we may expect, the odds of termination decrease as enrollment increases (baseline level of enrollment is *>* 1000).

**Table 3:** Maximum likelihood estimates of odds ratios and 95% profile likelihood confidence intervals of odds ratios

	Odds Ratio	Lower	Upper
enrollment_group 0–100	3.96	3.47	4.54
enrollment_group 101–1000	1.56	1.36	1.80
enrollment_group Missing	1.23	1.00	1.51
T7 (Surgery)	1.90	1.74	2.07
T17 (Dermatological)	0.08	0.06	0.10
T12 (Coronary)	0.15	0.11	0.20
T8 (HIV/Pregnancy)	0.21	0.18	0.24

## Discussion

Our study demonstrates two important contributions of the use of latent Dirichlet allocation (LDA) in the analysis of unstructured data to predict clinical trial terminations. First, the use of the LDA generated topic probabilities in the predictive model enriched the analytical power of the model. The topic probabilities provided logical constructs with which to describe and present the characteristic relationships between the aspects of the corpora (study descriptions) and the outcome variable (clinical trial termination vs. completion). Other studies have highlighted such merits of the use of LDA in unstructured data analysis [9, 10].

In addition to enhancing the analytical framework, the use of LDA generated topics in predictive analysis also improved the predictive prowess of the model. As our findings section clearly shows, in the current predictive model, the topic probabilities outperform the structured research variables used for predicting trial terminations vs. completions. Moreover, the current model which used topic probabilities did outperform a prior predictive model that used text analysis but not topic probabilities [14].

## Conclusion

In this study, we set out to demonstrate that unstructured data can systematically employed to provide insight much like or in combination with structured data, in understanding why studies terminate or succeed. Follett et al. had a similar conjecture regarding the ability of using single terms (i.e., the presence or absence of selected terms). We identified the important terms using text mining techniques, and then dummy coded the important terms to use them together with structured predictor variables in a random forest model. Our previous analysis showed that the selected terms reinforced the overall predictive power of the model, but the contribution by the selected words was modest.

In our current analysis, we show that the use of topic modeling using LDA significantly raises the utility of unstructured data in better predicating the completion vs. termination of studies. Once the topic model probabilities are factored into the prediction the predictive potential of most of the structured data variables all but vanishes.

One notable thing that is observed in this study is the conceptual orderliness of the LDA generated topics as evidenced by their straight correspondence with meaningful labels. It is specifically noteworthy that most of the topic probabilities appear to portray the studies by the conditions that the they investigate (e.g., Topic 1 thru 3, Topic 5 thru 11, Topic 14 thru 24). Other topic probabilities relate to or highlight study design characteristics – such as study settings, study outcomes or other characteristics relating to the study itself. We believe that this result is indicative of the fact that the semantic structure of corpora are a better representative of the salient characteristic of the corpora than the labeling through the structured (factor) variables.

Our analysis of the relationship between topic probabilities and the “primary purpose” variable was partly motivated by our curiosity as to whether specific goals of a study disproportionately contributed to specific pattern of topic probabilities. The fact that the association between most of the topic probabilities and the “the primary purpose” variable were weak suggests that this might not be the case. We believe that in general, the reason why most of the structured variables were not fit for predicting study completion or termination is because the factors that underlay completion or termination are not exactly related to the documented characteristics the studies. The fact that we obtained promising results from our textual analysis approach therefore encourages us to pursue this line of work further.

The text mining approach that we implemented in the current study did not involve extensive data wrangling and transformations. This was deliberate in that at this initial stage of analysis, we wanted to preserve the basic elements of the text intact. We suspect that there is a good chance that the predictive approach may be improved by implementing some data pre-processing and variable transformations that are applied in standard NLP applications. As an example, filtering the most important terms using the inverse document term frequency weighting approach might help in maintaining model parsimony. In addition, stemming in order to convert words to their generic terms before creating the term frequencies may also add value to the modeling process.

The reason why we did not follow this approach was not exactly an oversight. At this stage of the investigation, we deemed it important to retain the original language structure used to describe the studies intact. In future works we plan to evaluate how feature selection and feature engineering can alter the results of prediction.

## Data Availability

The datasets generated and/or analyzed during the current study are available in (or could be extracted from) the clinicaltrials.gov repository, www.clinicaltrials.gov.

## Abbreviations

CTTI: Clinical Trials Transformation Initiative
FDAAA: Food and Drug Administration Amendments Act (2007)
FDAMA: Food and Drug Administration Modernization Act (1997)
GLM: General Linear Model
HHS: Health and Human Services (Department)
LDA: Latent Dirichlet Allocation
LSI: Latent Semantic Indexing
ML: Machine Learning
NIH: National Institute of Health
NLP: Natural Language Processing
NSF: National Science Foundation
ROC: Receiver Operating Characteristic (curve)
